# The impact of improved data quality on the prevalence estimates of anthropometric measures using DHS datasets in India

**DOI:** 10.1038/s41598-021-89319-9

**Published:** 2021-05-21

**Authors:** Harsh Vivek Harkare, Daniel J. Corsi, Rockli Kim, Sebastian Vollmer, S. V. Subramanian

**Affiliations:** 1 Centre for Modern Indian Studies (CeMIS), Georg-August University Göttingen, Göttingen, Germany; 2 Faculty of Medicine, University of Ottawa, Post – 501 Smyth Road, Box 241, Ottawa, ON K1H 8L6 Canada; 3 Division of Health Policy and Management, College of Health Science, Korea University, Seoul, South Korea; 4 Interdisciplinary Program in Precision Public Health, Department of Public Health Sciences, Graduate School of Korea University, Seoul, South Korea; 5 Department of Social and Behavioral Sciences, Harvard T.H. Chan School of Public Health, Boston, MA USA; 6 Harvard Center for Population and Development Studies, Cambridge, MA USA

**Keywords:** Biomarkers, Epidemiology

## Abstract

The importance of data quality to correctly determine prevalence estimates of child anthropometric failures has been a contentious issue among policymakers and researchers. Our research objective was to ascertain the impact of improved DHS data quality on the prevalence estimates of stunting, wasting, and underweight. The study also looks for the drivers of data quality. Using five data quality indicators based on age, sex, anthropometric measurements, and normality distribution, we arrive at two datasets of differential data quality and their estimates of anthropometric failures. For this purpose, we use the 2005–2006 and 2015–2016 NFHS data covering 311,182 observations from India. The prevalence estimates of stunting and underweight were virtually unchanged after the application of quality checks. The estimate of wasting had fallen 2 percentage points, indicating an overestimation of the true prevalence. However, this differential impact on the estimate of wasting was driven by the flagging procedure’s sensitivity and was in accordance with empirical evidence from existing literature. We found DHS data quality to be of sufficiently high quality for the prevalence estimates of stunting and underweight, to not change significantly after further improving the data quality. The differential estimate of wasting is attributable to the sensitivity of the flagging procedure.

## Introduction

**Figure 1:**
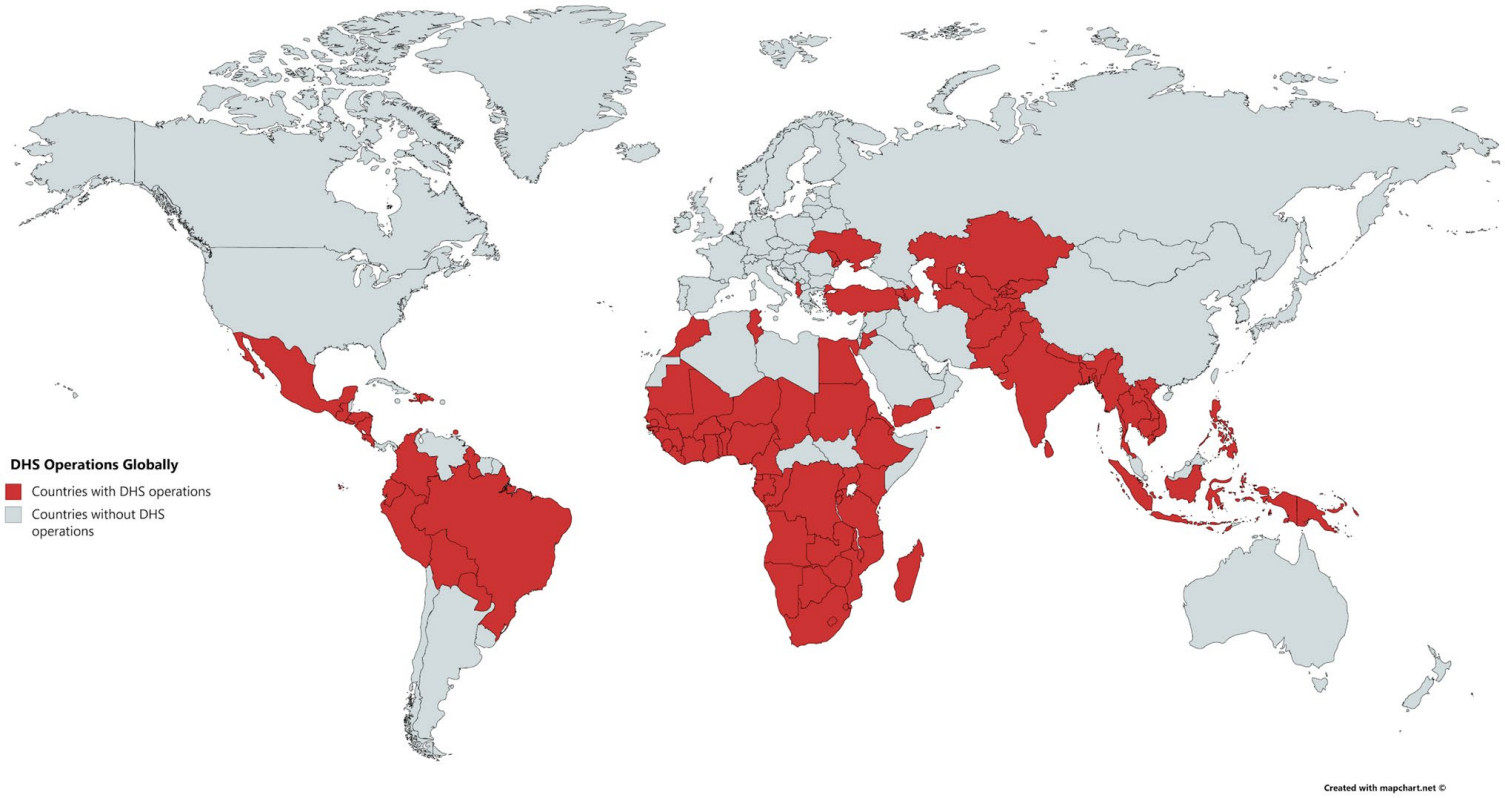
DHS operations globally. Source: https://dhsprogram.com/Countries/index.cfm?show=countries; map created at www.mapchart.net.

The anthropometric measurements of height and weight are used to calculate anthropometric failures of stunting, underweight, and wasting, all of which are fundamental indicators of childhood health and nutrition. When considering the prevalence of malnutrition and undernutrition in a population, the pervasiveness of these anthropometric failures is taken into consideration, and used to assess malnutrition and track progress in the SDGs. Population-based surveys such as those conducted by the Demographic and Health Surveys (DHS) are considered reliable data sources for arriving at these indices^1^. The DHS collects information on a range of health, socio-economic, demographic, and anthropometric variables, giving us an overview of the country’s health status. The program collects data from 102 countries worldwide, as can be seen in Fig. 1, which further stresses the importance of adequate data quality. The DHS database is open-access, making it an extensive repository of anthropometric data from LMICs. This ubiquity of the DHS data also necessitates a rigorous check on their data accuracy. The accuracy and precision of the survey data used to correctly determine children’s nutrition status in low-income countries is what subsequently determines further policy initiatives in that field.

Globally, around 149 million children, or 21.9% of all children were found to be stunted in 2018^2^. Of these 149 million children, close to 47 million live in India alone. India thus accounts for roughly a third of the world’s total number of stunted children. In this context, focussing our study and analysis on India becomes all the more relevant. Prevalence estimates of anthropometric failures have been found to differ across regions, surveys, and studies. Onis et al. report the share of stunted children in Africa to be 37.6% in 2015^3^. At the same time, a study by UNICEF reports stunting for the same region to be 32.0% at the end of 2014^4^. Such inconsistencies can often prove challenging for policymakers and can lead them to question the reliability of the data. These discrepancies are also problematic, raising doubts over which prevalence estimates to be used for policy-making.

Standardization of data quality checks is one possible method of ensuring quality and consistency^5^. In spite of the various available guidelines for achieving accuracy in anthropometry measurements, it still remains a complex task prone to errors. There are a variety of possible problems which could occur at multiple stages throughout the survey process which can be classified into two broad types—repeated measures which return the same value, and diverging measurements that depart from the true value^6^. While the former gives inaccurate and biased estimates, the latter returns imprecise and varying values. The sources of variation can be found in the imperfect use of measurement instruments, technician’s bias, inaccurate data-entry, errors in analysis, handling of missing and improbable values, and so on. All of these different measurement problems can give rise to different problems with regards to the quality of data, such as implausibility with biological values, digit preferences, or excessive variance. Problems with the structuring and planning of surveys could lead to the improper representation of the actual base population. Such cases can be ascertained by looking at imbalanced age or sex ratios.

Further, the shape of the distribution (skewness and kurtosis) and the observed value of the standard deviation (SD) of the Z-scores are also important indicators of data quality. Given accuracy in Z-score calculation and measurements, we would expect the distribution to be symmetrical with a SD close to the expected value of 1.0 with respect to the reference distribution. Values deviating from 1.0 would imply a differential spread of the distribution from the reference population. Values higher than 1.3 would suggest inaccuracies due to measurement error. The WHO Global Database on Childhood Growth and Malnutrition has also given an expected range of SD of the Z-scores for the three anthropometric indicators of stunting, underweight, and wasting^7^:

- height-for-age Z-score: 1.10 to 1.30,
- weight-for-age Z-score: 1.00 to 1.20,
- weight-for-height Z-score: 0.85 to 1.10.

Grellety and Golden have also shown that the SD increases significantly with the share of random measurement errors in the dataset^8^. Thus, the magnitude of the SD, along with the number of missing, flagged, or implausible observations can serve as an indication of survey quality. Although there are numerous guidelines for assessing nutritional status of individuals already in place, the WHO in 1983 was the first to publish a guideline based on the use of z-scores^9^. Z-scores are measured in the SD units of a reference population.

The Standardized Monitoring and Assessment of Relief and Transitions (SMART) program was rolled out in response to the situation to implement a simplified and standardized method of conducting all parts of a survey ranging from planning and training to data analysis and reporting^10^. Since its implementation in 2002, there has been broad take-up of both SMART and its accompanying Emergency Nutrition Assessment (ENA) software^11^. The practice of performing plausibility checks to assess data quality, in particular, has increased.

Data quality can affect the prevalence outcomes via a variety of channels. Excessive variation in the recorded observations could lead to unreliability as indicated by an unusually high or low SD. A sample unrepresentative of the actual base population would have biased measurements of skewness and kurtosis and thus distort the true prevalence. Similarly, not accounting for technician effects such as digit preference could also lead to a biased estimate of the true value. Data quality can also contribute to the reduction of the measurement error, thus affecting the width of the estimation interval such that higher quality data would have a smaller estimation interval.

Finally, corrections to the variation in data can be done at many stages of a study. Data can be assessed and altered for misspecifications in the collection phase or can be cleaned out during the analysis stage. Alternatively, flags for unsuitable data quality can also be set in place during the data-cleaning or analysis stage. Furthermore, the SMART criterion introduces a different range of acceptable z-score SDs. The acceptable range for SD of WHZ is given as 0.85–1.20, with only a minimal penalty being imposed for values under 1.15.

The current study seeks to assess the differences in prevalence estimates of anthropometric failures caused by a change in the quality of data. We identify the differences in these estimates as generated by the flagged and unflagged set of observations. More specifically, the study seeks to derive the changes in these estimates by excluding unreliable low-quality data. As a next step, the paper attempts to explain this differential impact of increased data quality. As a novel approach, we also try to establish whether these changes in data quality are driven by income levels by conducting a subnational analysis of 36 Indian states and union territories. Our hypothesis is that richer states would have a higher standard of living in general which could contribute to less errant or extreme values.

## Results

**Figure 2:**
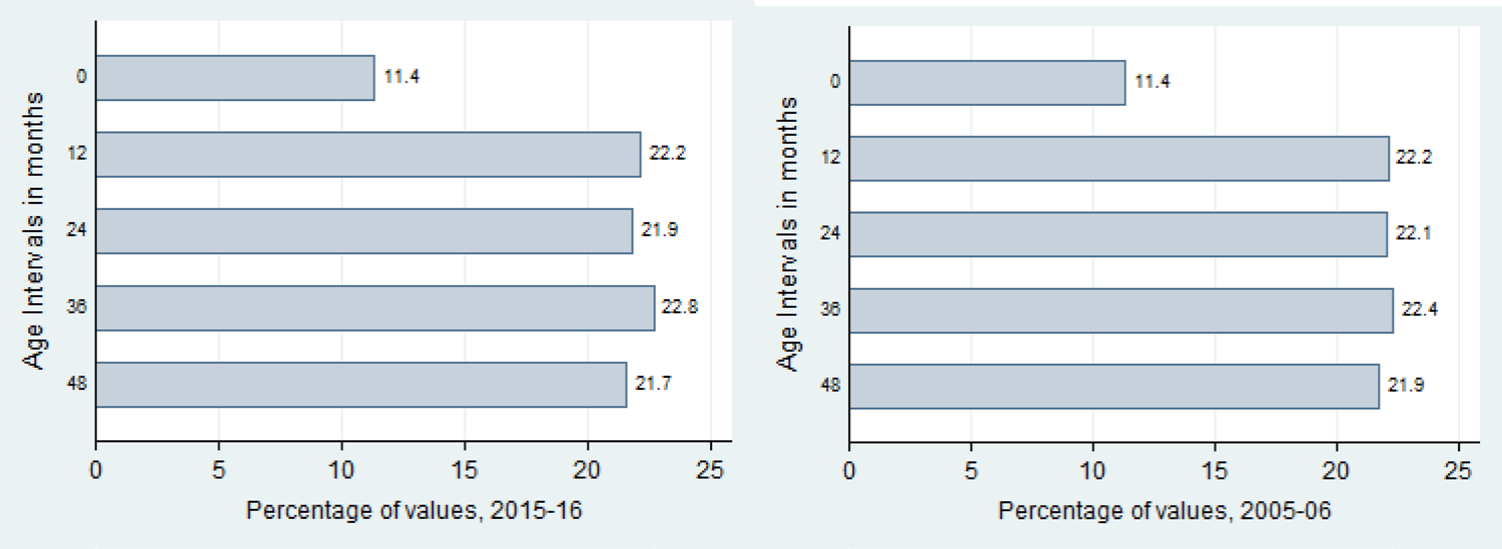
Age distribution in completed months.

**Figure 3:**
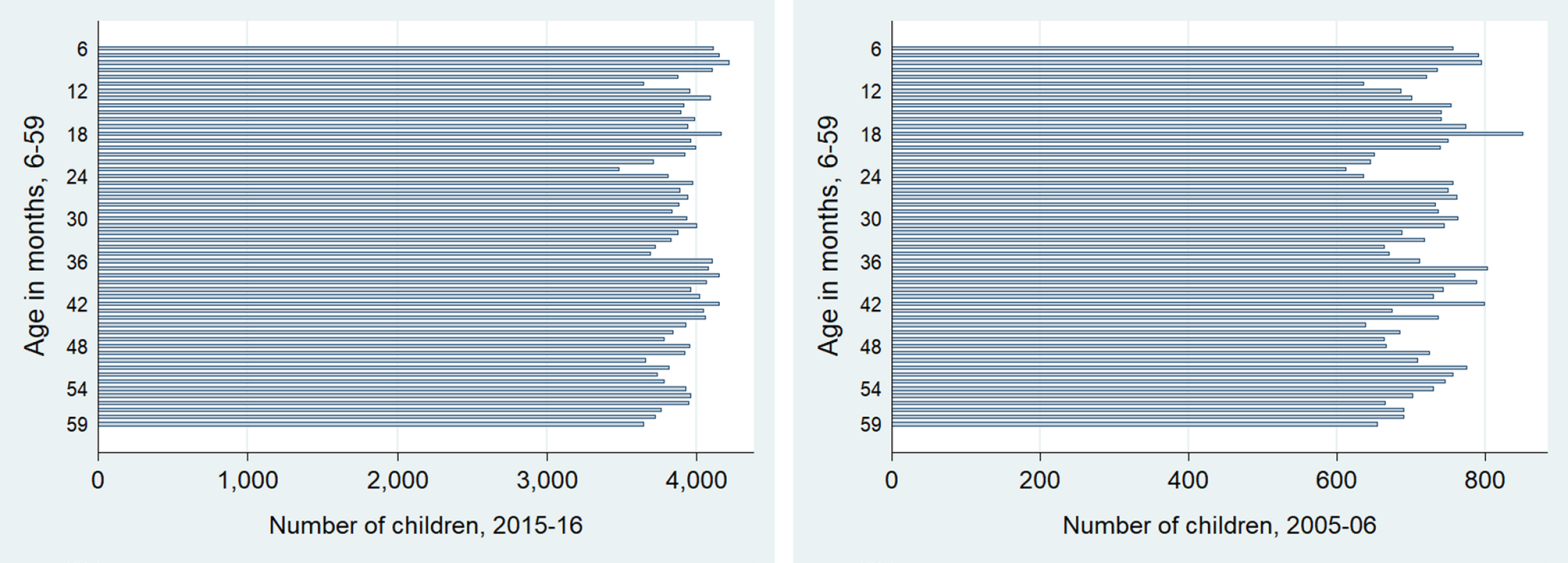
Age distribution in completed months.

The child age ratios for both DHS datasets was 0.81 which is very close to the expected distribution value of 0.85, as given by the SMART Methodology^12^. Additionally, the chi-squared test statistic for both datasets was not statistically significant, thus indicating that the variation in the data was caused by random differences and no systemic biases. The datasets thus serve as an accurate representation of the population and no flagging is done. Figure 2 further depicts the distribution of all observations into annual age intervals. For the age interval 0–11 months, only children aged 6–11 months are included. The number of children surveyed across each age group is close to 20% of all observations. Figure 3 similarly shows the age distribution of all observed children in completed months. Visual inspection is suggestive of the fact that while both datasets have a good coverage of children across all ages, the 2015–2016 dataset is somewhat more inclusive and extensive. This could be due to its higher sample size, however. Both Figs. 2 and 3 are used to assess age heaping in the dataset.

The sex ratio for both 2005–2006 and 2015–2016 dataset was 1.08 which is very close to the national sex ratio of India (1.07)^13^. Furthermore, a chi-squared test of the observed overall sex ratio against the expected one returns an insignificant chi-squared test statistic, meaning that the variation in data is also due to random differences. Flagging is thus also refrained from here.

Table 1 presents the cut-off points and percentage of values considered implausible with life according to the SMART flags and the WHO Child Growth Standards. Assuming all anthropometric z-score values lying outside the WHO flagging range to be due to inaccuracies in anthropometric measurement or in recording of the data, we deduce that WAZ measurements are the most precise (less than 0.40% implausible values for both datasets). This is followed by HAZ measurements (1.49% and 2.44% implausible values for the 2005–2006 and 2015–2016 dataset respectively) and lastly by the weight-for-height z-score measurements (2.13% and 2.15%). These observations, whether due to an error in collection or recording, are deemed implausible with life and are thus flagged accordingly. According to the WHO, a percentage of implausible values exceeding 1% is indicative of poor quality data^14^. Also important to note here is that while a high number of flagged observations indicates poor data quality, a lower percentage of the same doesn’t necessarily imply good quality as inaccuracies in measurement can still lead to values which are within the WHO-specified range.

**Table 1:** Cut-off values for acceptance for SMART and WHO flags and percentage of excluded observations.

	Smart				WHO			
	2015–2016		2005–2006		2015–2016		2005–2006	
	Cut-off	%	Cut-off	%	Cut-off	%	Cut-off	%
HAZ	− 4.65 to 1.54	8.34	− 4.96 to 1.23	8.93	− 6 to 6	1.49	− 6 to 6	2.44
WAZ	− 4.67 to 1.52	2.52	− 4.79 to 1.40	2.78	− 6 to 5	0.32	− 6 to 5	0.35
WHZ	− 4.09 to 2.10	6.04	− 3.99 to 2.20	5.68	− 5 to 5	2.13	− 5 to 5	2.15
Overall	–	12.32	–	12.05	–	3.50	–	3.11

Table 2 presents a brief summary of the DPS of height and weight measurements for the NFHS-3 and NFHS-4 datasets. As we can see, all scores are below 20, indicating a low level of digit preference in all datasets. Height measurements also appear to be more inaccurate as compared to weight measurements.

**Table 2:** Digit preference scores for height and weight measurements of the NFHS-3 and NFHS-4 dataset.

	2015–2016	2005–2006
Height	13.32	19.87
Weight	4.17	2.09

We apply the Standardized Monitoring and Assessment (SMART) flagging criteria to our analysis^10^. The SMART flags, as aforementioned, are based on statistical plausibility and are rather flexible as opposed to the WHO’s fixed flagging criteria. This means that the range of acceptable z-score SD would be based on the observed mean. Applying the SMART flags to our datasets leads to a loss of 12.05% and 12.32% of observations in the 2005–2006 and 2015–2016 dataset, respectively. A detailed loss of observations by specific z-scores can be seen in Table 1. To further analyze the quality of the unflagged data, we assess its z-score and normality, which is presented in Table 3. The z-scores for height-for-age and weight-for-age measurements are used for estimating the prevalence of stunting and underweight and are both within the range specified by the WHO 2006 Child Growth Standards.

**Table 3:** Z-score normality assessment of the unflagged data.

	HAZ		WAZ		WHZ	
	2015	2005	2015	2005	2015	2005
Post-flagging mean values (SD)	− 1.64 (1.27)	− 1.87 (1.30)	− 1.57 (1.04)	− 1.67 (1.07)	− 0.93 (1.14)	− 0.83 (1.08)
Skewness	0.09	0.00	0.04	− 0.03	− 0.12	− 0.88
Kurtosis	2.59	2.52	2.79	2.71	2.88	2.92

Coming to the weight-for-height z-score, it is important to note that there is a discrepancy in the accepted range for the WHZ as the WHO 2006 Child Growth Standards and the SMART Methodology differ here. WHO imposes a penalty for WHZ over 1.1, while SMART does that for values over 1.2. In a study published by Golden and Grellety, they study data from over 200 surveys to arrive at the conclusion that the data is almost always normally distributed and had standard deviations between 0.8 and 1.2^15^. We thus choose to go by the SMART criteria and set our acceptable range for SD as 0.8–1.2. Thus, the NFHS-4 WHZ value of 1.14 is acceptable with only a minimal penalty. The SMART Methodology further reports that for surveys with SD less than 1.2, data can be taken to be of sufficient quality to report the observed prevalence^12^.

### Estimating anthropometric failures

Prior to any flagging, the prevalence estimates of stunting, underweight, and wasting for India according to DHS data stood at 46.57%, 39.27%, and 18.46% respectively for the years 2005–2006. The same estimates for 2015–2016 stood at 40.01%, 36.04%, and 20.59% respectively. Table 4 gives an overview of prevalence estimates based on our analysis both before and after flagging. The changes in prevalence estimates is prominent only for the anthropometric failure of wasting. There is only a very small decrease in the estimates of stunting and underweight for both years.

**Table 4:** Prevalence estimates of anthropometric failures before and after flagging.

	2015–2016		2005–2006	
	No flags (%)	Post flagging (%)	No flags (%)	Post flagging (%)
Stunting	40.01	39.74	46.57	46.19
Underweight	36.04	35.72	39.27	38.86
Wasting	20.59	18.03	18.46	16.22

### Socioeconomic and demographic characteristics

**Figure 4:**
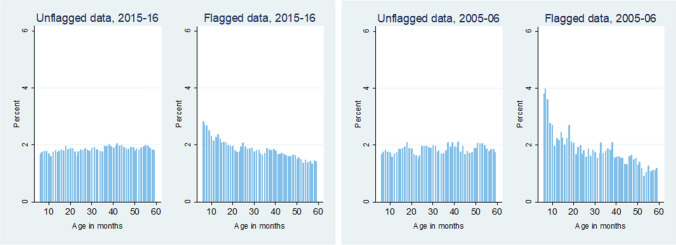
Differences in distribution of ages for flagged and unflagged data.

When checking for the drivers of data quality, we look at the sex, age distributions, wealth indices, and the size of the household for unflagged (good quality) and flagged data (poor quality). The sex ratios for both datasets flagged and unflagged are very similar to each other. Sex thus doesn’t seem to be a source of differential data quality. Figure 4 plots the distribution of children’s monthly ages for all flagged and unflagged observations. While the unflagged data has an equal distribution of all ages, the flagged observations have an unequal distribution of ages. This could imply that the variation or inaccuracies in measurements are coming from younger aged children. The same is seen for the 2015–2016 dataset as well.

**Figure 5:**
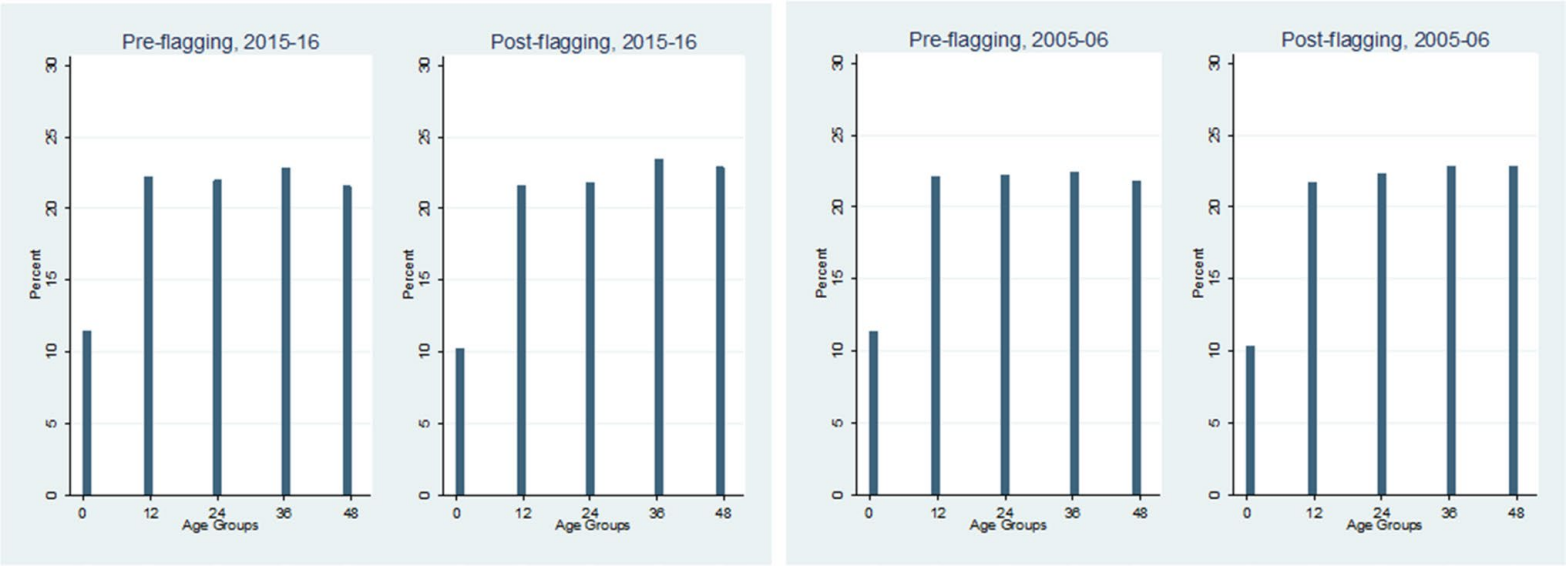
Pre- and post-flagging age distribution in groups of 12 months.

To ensure that our post-flagging dataset is not unequally distributed among the different age groups, we also do a comparison between the pre- and post-flagging age distribution, the results of which are shown in Fig. 5.

Figure 5 tells us that even after the flagging procedure, the overall distribution of age ratio remains relatively constant, thus ensuring a proper representative sample for the chosen population.

**Figure 6:**
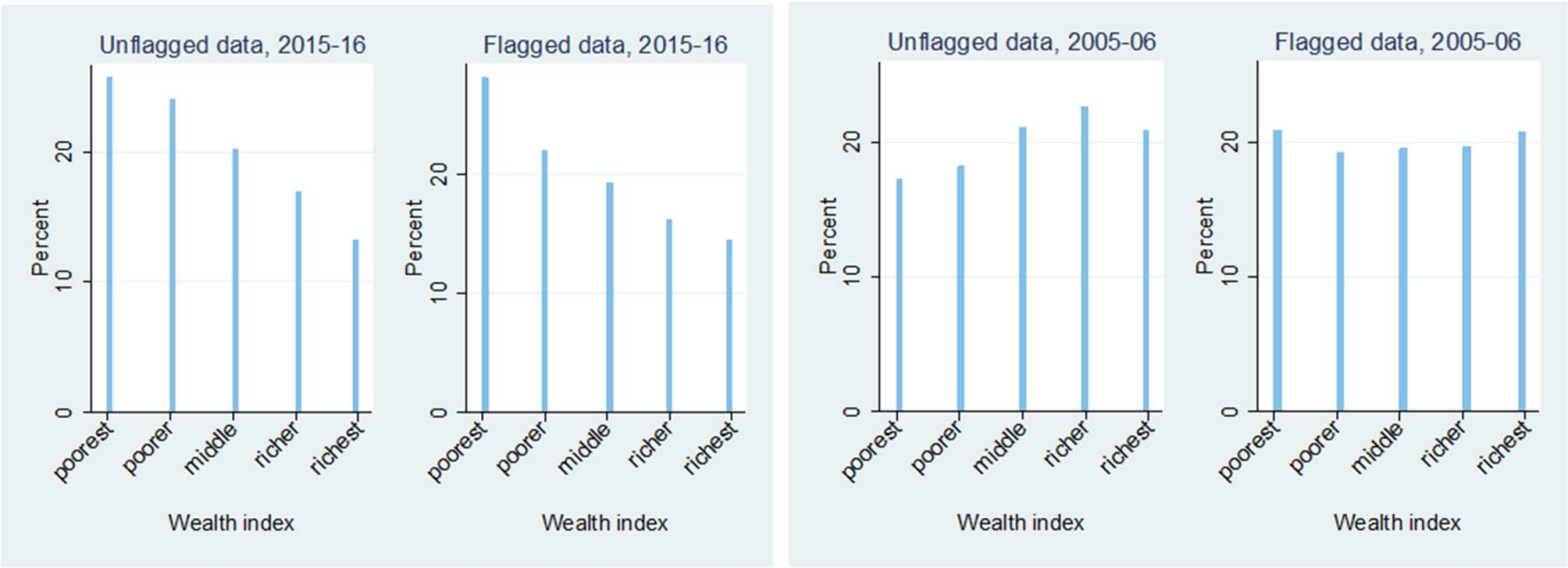
Differences in distribution of wealth indices for flagged and unflagged data.

Plotting the distribution of wealth indices for both datasets in Fig. 6, we see that there are no substantial differences between the flagged and unflagged observations. There is a similar pattern within the datasets indicating that wealth of the household doesn’t affect the quality of data.

**Figure 7:**
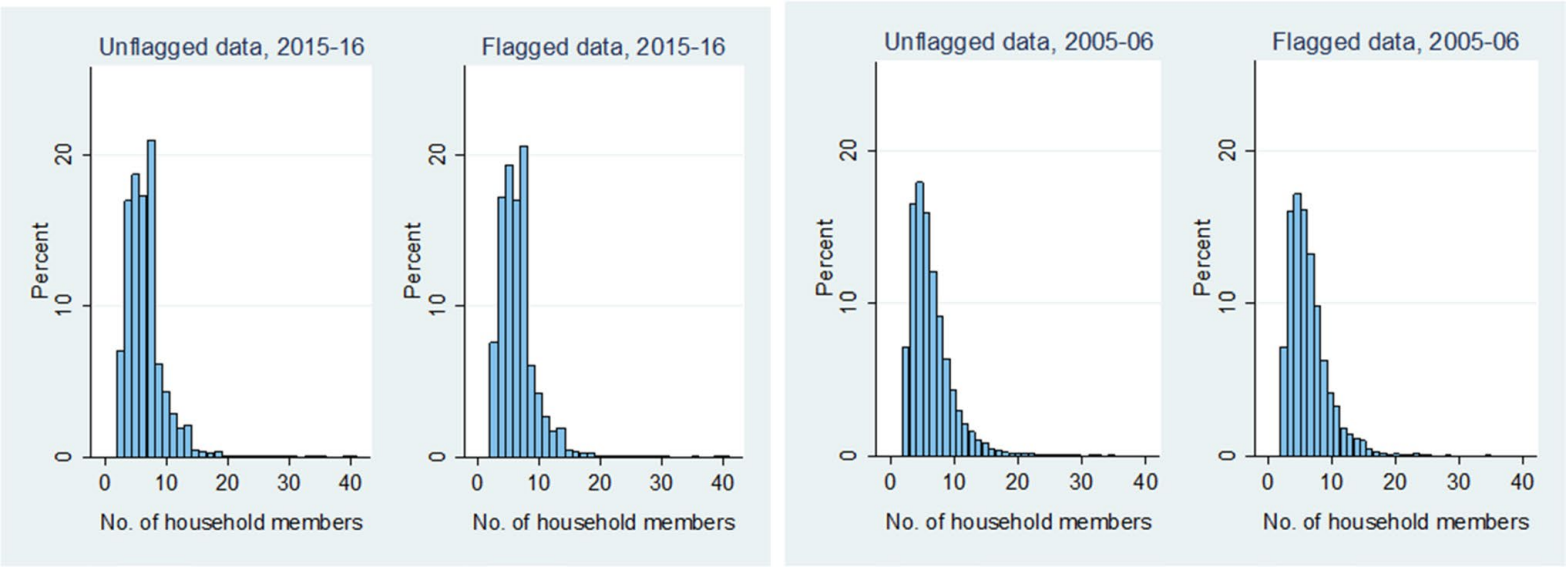
Differences in distribution of household members for flagged and unflagged data.

For the number of household members, there is no visible change in the distribution for flagged and unflagged values for either dataset, as can be seen in Fig. 7.

### Subnational analysis

Looking into the socio-demographic and geographical drivers of missingness, we find that after adjusting for the total number of observations in each quintile, the two poorest quintiles had slightly higher shares of the total number of missing values. In comparison, the three richer quintiles had a lower share. There is also significant variation in the percentage of missing observations by the Indian states. The states of Uttar Pradesh, Bihar, and Madhya Pradesh have a missingness of 9–17% compared to the national average of 8%. Incidentally, these also happen to be some of the poorest states of the country. It must also be noted that these states make up for the largest share of observations in the whole dataset, and so a larger share of missing observations is to be expected. In light of these findings, we ran a subnational analysis by income levels.

**Figure 8:**
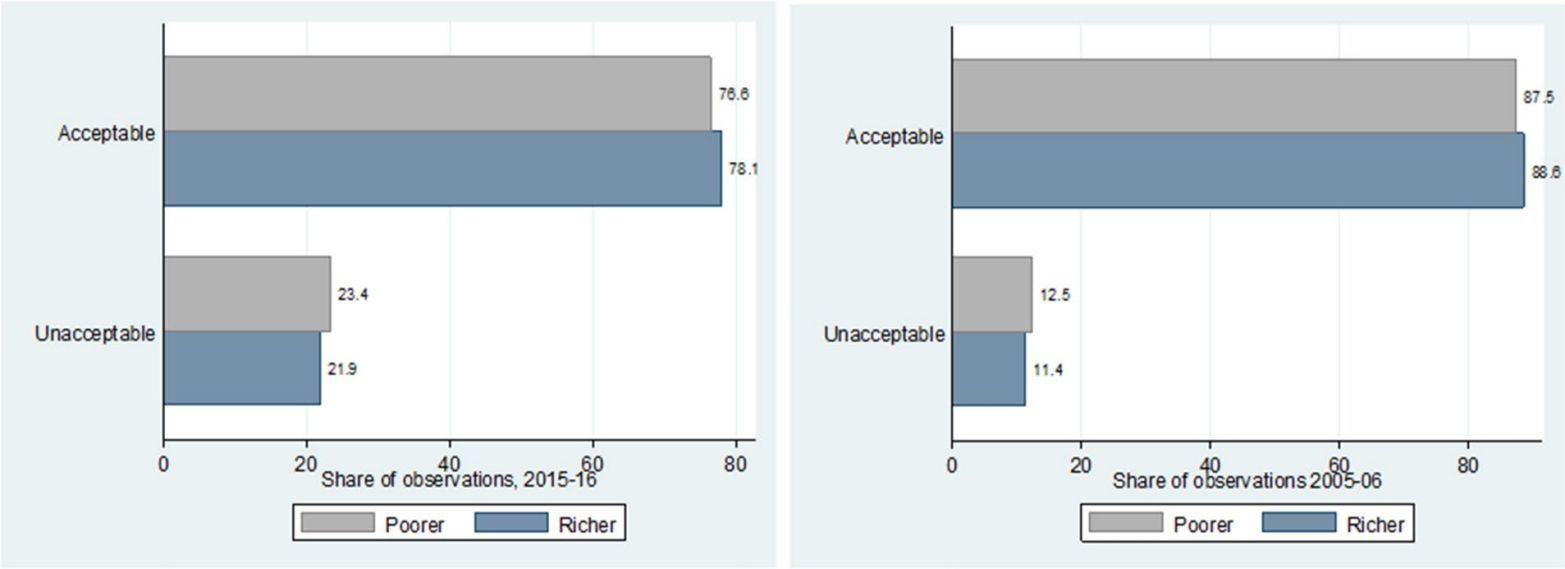
Variation in data quality for Indian states based on their wealth.

What we see from Fig. 8 is that richer states perform better in terms of the quality of data generated in both NFHS survey rounds. The difference however, is only marginal.

**Figure 9:**
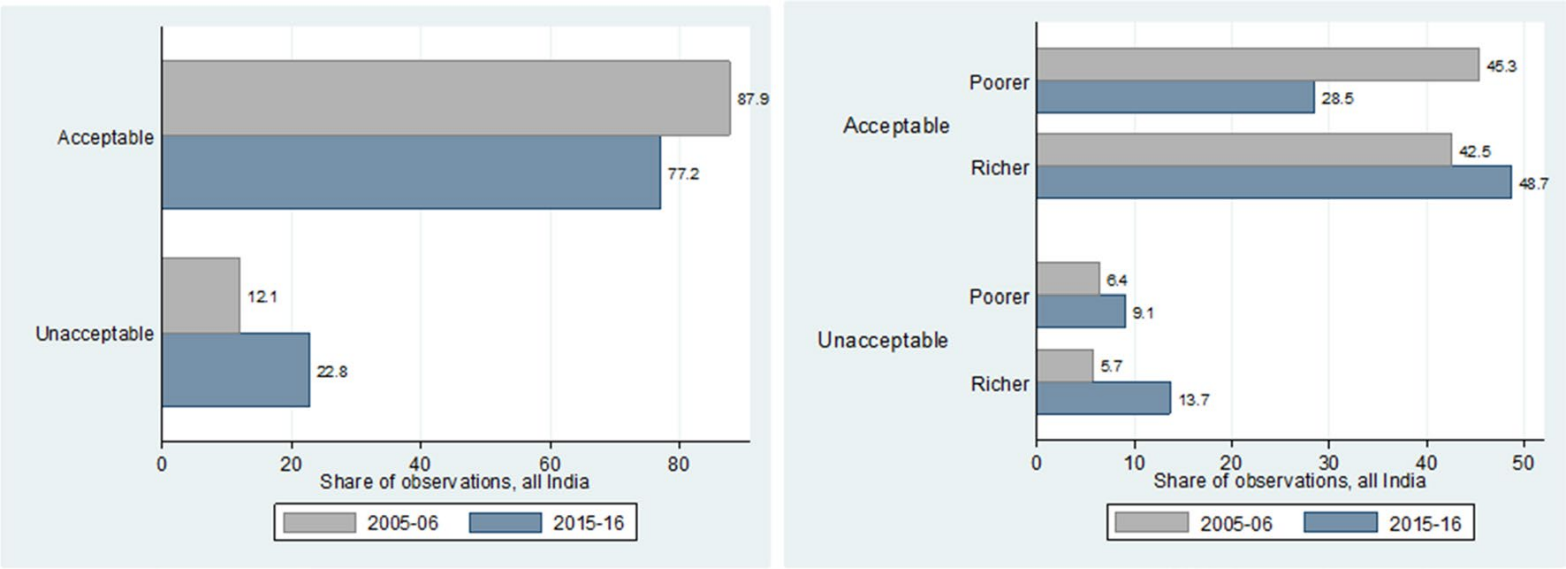
Variation in data quality over time for India overall and by wealth of states.

When looking at the variation in data quality over time for all of India, we see from Fig. 9 that there has been a significant decrease in the share of high-quality data for the latter round of the survey in 2015–2016. This could however, also be simply due to the fact that the 2015–2016 dataset has more observations, thus making it more difficult to manage data quality. On the other hand, we also see that richer states saw somewhat of an improvement over time in their share of high-quality data. Poorer states however, had a larger decrease in the share of high-quality data, which might perhaps explain the overall decrease in data quality over time.

## Discussion

This study provides an in-depth analysis and assessment of data quality in the NFHS and its subsequent effect on the prevalence estimates of anthropometric failures. We find that stunting and underweight estimates undergo a minimal to insignificant change while the estimate of wasting falls by around 2 percentage points. We aimed to show the impact data quality can have on the desired results. From the analysis conducted for this study, we find the data quality of the DHS India surveys (NFHS-3 and NFHS-4) to be acceptable for all of our chosen data quality indicators, except for biologically implausible values. This is an important finding in itself as it confirms the quality levels of DHS datasets. These may also be generalized to other DHS datasets with due discretion. However, since DHS are conducted independently by different countries, data quality differences might still arise. Identifying the true impact of data quality on prevalence estimates of anthropometric failures could potentially free up resources for more novel and fruitful avenues of research otherwise invested in improving data quality.

For the two DHS India datasets analyzed in this study, the sex ratios were balanced. There was no evidence of substantial age heaping, and we addressed the issue of digit preference bias. The normality assessment done post-flagging was also in line with the general good data quality indicators. However, applying the SMART flags, led to an overall loss of 12% of all observable values from the datasets of 2005–2006 and 2015–2016, respectively.

Our main results of a change in the prevalence estimates of anthropometric failures as captured in Table 4 shows a less than 0.5 percentage point change in the prevalence estimates of stunting and underweight before and after flagging. This indicates that flagging observations with poor quality or questionable values did not substantially change estimates of anthropometric failures. In other words, improving data quality further did not make a big difference in the desired end result of estimating stunting and underweight prevalence. However, coming to the estimate of wasting, we see a fall in the estimated number of children with wasting by a little over 2 percentage points.

The differential impact on the estimate of wasting can be understood by looking at the range of z-score values. Since the SMART flags are flexible flags based on the mean z-scores of a population, the number and magnitude of values to be included and excluded in the SMART flags is sensitive to the mean. In our analysis, the mean weight-for-height z-score (WHZ) is − 0.89 and − 0.99 for the 2005–2006 and the 2015–2016 dataset, respectively. This is higher than the means of height-for-age and weight-for-age z-scores of both datasets, which would imply that the lower end of the SMART flag’s acceptable values would also be higher for WHZ. This leads to a larger share of values on the lower end of the WHZ distribution being excluded post-flagging. We thus get a lower estimate of wasting than that before flagging. If, on the other hand, the study had more observations on the lower end of the distribution, we would observe a lower mean z-score value and a higher estimate of wasting. This differential impact of wasting can thus be explained by a relatively larger share of children on the higher end of the WHZ distribution. Incidentally, children on the higher end of this distribution (WHZ > 2) are classified as overweight^7^. The share of overweight and obese children in India was 19.3% in 2010^16^. This sensitivity of the SMART flagging procedure to the means of z-scores could then lead to the exclusion of observations at the lower end of the distribution and thus lower estimates of wasting. As a supplementary exercise, we analyse the effect of a more inclusive WHZ on the prevalence of wasting, which does indeed suggest that using a WHZ range more inclusive of the lower end of the distribution removes any differences seen in the prevalence estimate of wasting post-flagging. Furthermore, Grellety and Golden have discovered empirical evidence of having more exclusive flags resulting in lower prevalence estimates due to the inclusion of lesser extreme observations^17^. Moreover, Corsi et al. found in a simulation analysis aimed at isolating and identifying the effects of differing anthropometric measurements on the prevalence of stunting, underweight, and wasting that inaccuracies in weight at the level of 0.1 kg could result in a 2% overestimation of the prevalence of wasting^18^. This analysis aimed to determine the extent and impact of different anthropometric measures and their distributional properties on prevalence estimates. This suggests that prevalence estimates of anthropometric failures are sensitive to the distribution and quality of observations and can be significantly affected by it. This further underlines the importance of establishing and achieving a standardized survey procedure to ensure reasonable data quality.

Looking at the drivers of low-quality data, we see no significant changes between the socioeconomic characteristics of flagged and unflagged data except for children’s age distribution. The analysis shows that most flagged observations with poor data quality had a higher share of values from the younger end of child age distribution. Within the younger end of the distribution, most flagged observations are for children aged under 12 months. It should be noted here that younger children, especially those under 6 months of age, are difficult to measure accurately. For children under 24 months height is substituted for by a recumbent length, and a special method is applied by the technicians to measure the length accurately. One of the technicians presses down on the child’s knees so that the limbs are fully extended. This procedure further highlights the importance of technician training in accurately measuring anthropometric data.

Previous studies have already documented that children under 2 years of age have more variant measures. A study published by the DHS shows that the SDs for WHZ and HAZ (both measures are dependent on height) are almost always higher for children under 2 years of age, which could perhaps be due to higher agitation of younger children^19^. Another study has also shown that most growth faltering in the Indian context takes place before a child has achieved 24 months of age^20^. So while this is an important issue, it is not the most pressing one given that the age distribution of children before and after flagging remains relatively constant, as can be seen in Fig. 6. We are thus assured of a representative and complete sample. Nonetheless, there is a dire need for novel improvements in measurement methods for this age cohort to improve the overall level of DHS data quality. Specialty training can also be given to technicians just for this regard.

Grellety and Golden have stated that survey SDs are often drivers of change in estimation prevalences via the effect of random errors^17^. They also argue the superior accuracy of SMART flags over WHO-flagging. It should however be noted that the WHO itself has expressed concern over the ideal SD range as suggested by the WHO 1995 Technical Report on account of the following^21^: (1) The report relied on a set of surveys of which not all were nationally representative and were in fact conducted via the rapid nutrition surveys for emergency situations. This could lead to a more-than-normal homogenous population. (2) The range was based on the distribution of z-scores calculated using the NCHS/WHO reference for child growth standards. (3) The actual flagging system used by the 1995 technical report was a lot more conservative than the WHO 2006 standards which would lead to narrower SD ranges. So even when the resulting SD follows the WHO-specified ideal range, it should not be taken as a touchstone for ensuring quality.

Our subnational analysis highlighting variations in data quality by income levels and over time lends a new perspective on the debate around DHS data quality. There has been a significant fall in the overall share of acceptable data quality over time for the DHS surveys. Furthermore, the fact that poorer states are lagging behind the richer ones, albeit by a small margin, is suggestive of further reforms needed in the method of measurement of anthropometric data. A possible explanation for the same is that since poorer states are generally also more unequal in terms of their income distribution^22^, there is simply more variation in the kind of nutrition every child receives in that state. What this means is that poorer states have a higher inherent variation in their anthropometric data and thus a higher SD. Further research would need to be undertaken to pinpoint the exact causes of these variations at lower income levels and then perhaps specialized training on the part of the technicians will be required to counteract these unfavourable forces. Alternatively, innovative methods of dealing with such inequality-induced variations can also be researched to account for the naturally larger SD range.

A strength of the study lies in its straightforward application and easy replicability of analysis, thus extending the limits of its applicability to datasets other than that of the DHS. Our research method uses multiple data quality indicators and flagging procedures to arrive at only the most ‘high quality’ observations. Deriving the prevalence of anthropometric failures from this pool gives us more credible and reliable results. A novelty of the paper was its subnational analysis looking at drivers of data quality within the Indian states. Income levels of different Indian states were found to have a minimal effect on data quality. The paper also looked at the evolution of data quality over time for India to highlight the changes in data quality. A limitation of the study is that while various data quality indicators are looked at and assessed, only a few are flagged to arrive at the differential impact in prevalence estimates made by them.

Further research could then possibly look at datasets with inherent flaws in quality indicators unassessed in our analysis (such as age heaping and sex imbalances) to strengthen and expand the argument of the study. Additional research could also be conducted to test the eventual generalization of the argument to other measures of anthropometric failures such as poor mid-upper arm circumference. Further research is also warranted towards exploring novel methods of accurate anthropometric measurements for younger children in order to avoid unnecessary loss of data. The same is true for dealing with anthropometric data from poorer regions within a country. Lastly, improved technician training with a focus on more precise data collection for younger children and for poorer states could also prove helpful in this regard.

## Methods

Our data originates from the 2005–2006 to 2015–2016 National Family Health Surveys (NFHS) with measurements taken from distinct samples^23^. The NFHS follows the same standards and procedures as prescribed by the DHS and can hence be taken to be equivalent. A summary of the data we use can be found in Table 5. These are large-scale household surveys with a targeted sample group of women aged 15–49 years and their children aged 0–60 months. The surveys are based on nationally representative sampling plans of households. The surveys cover data on a broad range of nutrition and health indicators, including child mortality and anthropometric measurements. They also include socioeconomic indicators such as household wealth and education of respondents. Since 2005–2006, the NFHS survey protocol includes the anthropometric measurements of all children under the age of five listed in the Household Questionnaire, and not just those born to the women interviewed in the Women’s Questionnaire. This was done to ensure proper representation of all children^24,25^.

**Table 5:** Summary of the data used.

	2015–2016	2005–2006
N (share of total data)	259,627 (83.43%)	51,555 (16.57%)
Mean age in months (SD)	29.66 (17.15)	29.54 (17.21)
Mean height in centimetres (SD)	83.38 (14.01)	82.36 (14.30)
Mean weight in kilograms (SD)	10.46 (3.32)	10.33 (3.38)

In this study, we deal with age, height, and weight measurements. The number of children eligible for measurements was 51,555 for the 2005–2006 dataset (NFHS-3), and 259,627 for the 2015–2016 dataset (NFHS-4) thus bringing the total number of children studied to 311,182. The number of children aged 0–5 years according to the 2011–2012 census of India was computed to be 138,861,008, thus putting our total sample size to be at around 0.2% of the entire population. While this can appear to be a small number, previous research has provided evidence that it is more than enough. Krejcie and Morgan^26^ have shown how for populations above 1 million, the number of observations needed increases at a diminishing rate and stays rather constant at around 384 observations. It is important to remember here that the NFHS data can be representative down to the district-level. Here, we run a simple cross-checking exercise to see which of the states contribute to the sample size by less than 384 observations. We find that for NFHS-4, it is only the state of Goa and the union territories of Chandigarh and Lakshadweep that have less than the required number of observations. Barring the three, our data has enough observations to be duly representative. NFHS-3 had enough observations for all states and union territories covered in the study.

Furthermore, we also assessed the ideal sample size for a given population with the following formula, where N is the population, z the z-score, and e the margin of error^27^.

$${\text{Sample}}\;{\text{size}} = \frac{{\frac{{z^{2} \times p(1 - p)}}{{e^{2} }}}}{{1 + \left( {\frac{{z^{2} \times p(1 - p)}}{{e^{2} N}}} \right)}}.$$

So for a population of 138,861,008, the sample size needed at a 99% confidence level and 1% margin of error is about 16,640. In this scenario, both of our datasets of NFHS-3 and NFHS-4 have more than enough observations to qualify for a representative sample population.

Age is assessed by self-reports and cross-checked with the completion of reproductive calendars during household visits. Standard DHS protocols also include the training of field investigators who are instructed to weigh each child using a solar-powered digital scale (Seca 878) with a precision of ± 100 g^28^. The SMART Methodology also propagates the use of digital scales as opposed to salter or hanging scales since salter scales are a lot more cumbersome to handle and require more fine-tuning on part of both the technician and the child being measured. For children under 2 years of age, tared weights. Vertex height or stature is measured standing for children above two years and lying or recumbent length for those under 2 years. When accurate age cannot be obtained, lying length is measured for children less than 85 cm, and stature if equal to or larger than 85 cm. An adjustable measuring board is used for the same with a theoretical accuracy of 1 mm^19^. In the absence of digital scales, the WHO mandates ensuring that the child’s eye is parallel to the footpiece, i.e. the child’s head is in the Frankfort horizontal plane^29^. When measuring recumbent length for children under two years, the technician is instructed to lightly press down on the child’s knees to ensure accuracy in measurement. The technician also notes down whether lying length or standing stature was recorded.

A potential serious drawback to the Seca scales is that the ground must be completely flat at the time of measurement. If the technicians are not properly trained to ensure this requirement, there could be errors in measurement to the extent of 0.10 or 0.20 kg. This further goes to highlight the important role of technician training when it comes to conducting surveys. As of now, DHS technicians are trained to measure height/length and weight according to the internationally recommended standard protocol of ICF International 2012^19^.

Nutrition status is assessed using data surveys including anthropometric measurements from a representative sample of children aged 6–59 months, especially for regions under nutritional stress^17^. For the initial data cleaning, we dropped observations of all children with any of the following criteria:

1. age < 6 months (n = 27,315, equal to 8.78% of the total data),
2. Missing, or instances where the child was not present or refused to take measurements (n = 28,775, equal to 9.25% of the total data).

Further, we restrict our sample to only singleton births as the WHO growth standards are otherwise inapplicable (n = 3382, or 1.09% of the total observations dropped). After this data cleaning, we have N = 251,710 observations. We also study the drivers of missingness as a part of our analysis.

Ulijaszek and Kerr present the hierarchy in the precision of anthropometric measurements, with weight and height measures considered to be the most precise^6^. We thus focus on these to derive the prevalence of stunting, underweight, and wasting. Any quality assessment of and based on these measures can thus be considered a good quality indicator for all other anthropometric variables in a dataset. Accuracy in age measurements is also crucial for estimating correctly the measures of anthropometric failures of stunting (height-for-age) and underweight (weight-for-age). In general, it is easier to estimate the month of birth for younger children. Moreover, the SMART methodology has outlined further practices that could be used to estimate correctly a child’s age^10^. In emergency situations however, age is mainly used to determine the inclusion or exclusion of a child from the survey, and whether the sample has an equal distribution of children across ages. This is because age is not used to calculate wasting prevalence (weight-for-height), which is what is used by policymakers for emergency situations.

All Z-scores (height-for-age, weight-for-age, and weight-for-height) were calculated in accordance with the WHO Child Growth Standards^7^. The WHO Growth Standards are based on a sample of healthy children from six different countries on five different continents living in an environment that did not constrain optimal growth, and are different for boys and girls. The z-scores are thus a function of age and sex of the child.

It is also important to note the training of the technicians in this regard, since a lot of data variation can be attributable to malpractices on their part. The DHS has thus mandated technician training via the use of its published manual titled ‘DHS Interviewer’s Manual’^28^ which trains the technicians on a range of practices from conducting an interview to fieldwork procedures. Furthermore, supervisors are also given standardised guidelines for technician training and skill assessment with the ‘Training field staff for DHS surveys’ manual^30^.

Our first data quality indicator is the child age ratio. This is calculated as the number of children aged 6–29 months over the number of children aged 30–59 months. Ideally, the expected ratio should be close to 0.85, given an equal coverage of all ages in the 6–59 month range^12^. Inherent variability however, is bound to occur due to recording error, uncertainty of a child’s date of birth, demographic changes, or due to variations in mortality rates over time^18^. Errors in age measurement thus can be of both types—repeated and diverging. Additionally, distribution of the ages of children 0–59 months is also calculated and presented to examine departures from the expected distribution (Fig. 3). We also conduct a chi-squared test for the goodness of fit for the overall expected age ratio as recommended by the SMART Methodology to find if the variation in the age ratio is due to random differences in sampling, or if a selection bias has influenced the data. In general, if the chi-squared test for the overall age ratio is not significant (as mandated by p > 0.05), then we can safely assume that the variation was caused by random differences^31^. However, it should be noted that there has been contention on the definition of age-heaping and its acceptable value, specifically on the differences in the month intervals between 6–29 and 30–59 months. The study therefore advises treatment of this data quality indicator with caution.

Our next data quality indicator is that of child sex ratio defined as the number of males to females in a given population. To ensure an equal representation of both sexes in a population, this ratio should be equal to 1.0. This however often differs due to reasons such as sex preferences and cultural norms, to name a few. In India specifically, cultural norms mandate a son preference, thus leading to female infanticide. This is what causes an inherent imbalance in the Indian sex ratio. We apply the same chi-square test and treatment to the child sex ratios as those towards the child age ratios as explained above.

Anthropometric z-score values improbable and implausible with life are accordingly marked with the WHO flags, according to the 2006 Child Growth Standards^29^.

We also choose to assess possible technician biases by accounting for digit preferences. A technician could have a conscious or a subconscious preference for a particular digit and may use it more or less than usual. Repeated application of the same could lead to the creation of a bias. A digit preference bias is thus, mostly a repeated measure form of error. The SMART Methodology suggests the use of a Digit Preference Score (DPS) to height and weight measurements individually, which is in turn derived from the WHO MONICA study^10,32^. The formula devised in the study desensitises the chi-squared test to allow for minor degrees of digit preference which are by themselves insufficient to significantly alter the results of the survey. Given no digit preference, we would expect an equal number of each terminal digit 0 to 9. This criterion is evaluated with the chi-squared test of the observed frequencies against the expected ones. The DPS is then evaluated on the basis of this chi-squared test statistic with the formula: DPS = 100 × (χ2/(df × N))½. Here, N is the number of total observations in the survey and df is the degrees of freedom. The scores range from 0 to 100 with lower scores denoting no digit preference and higher scores indicating a terminal digit preference capable of affecting the results of the survey. We calculate four such scores for each of the height and weight measurements of children in both datasets. Additionally, we also employ a visual inspection of the height and weight terminal digits as well as the Myer’s Blended Index as a supplementary exercise to further ascertain the terminal digit preference in DHS datasets.

The SMART flags are also applied after this to ascertain the prevalence estimates. Each individual anthropometric failure is flagged with the SMART flag for its own mean-specified range. A summary of all data quality indicators used is presented in Table 6. We’ve designed the analysis in a way that is comprehensive of different data quality indicators as well as straightforward in its application. This makes our analysis fairly easy to replicate.

**Table 6:** Summary of all data quality indicators used.

Indicator	Child age ratio	Sex ratio	Who plausible values	Terminal digit preference	Smart flag
Description	Percentage of children aged 6–29 months over 30–59 months	Number of males over number of females	− 6 < HAZ < 6	Visual inspection of digit preference and myer’s index	mean(z-score) ± 3.1
			− 6 < WAZ < 5		
			− 5 < WHZ < 5		

As a final step, normality assessment of the data is done by looking at the skewness, kurtosis, and SD of the z-scores. Skewness is a measure of symmetry or asymmetry and indicates the general direction of the data towards a particular magnitude, if any. Kurtosis is the mapping of the peakedness of a distribution and is indicative of its weighing. A perfectly normal distribution will have a skewness of 0 and a kurtosis of 3. It should be noted here that in countries with prevalent undernutrition, there would be a natural skew slightly to the left. Data quality in these cases can be assumed to be unreliable when the skew is excessive, i.e. lower than − 1 or greater than 1^33^. It should also be noted that our data concerns itself with a critical period of rapid physical growth which can negatively impact the normality of the data. It therefore must be dealt with caution. Similarly, sexual dimorphism in size and growth of humans can also impact normality. Even though our sample deals with a much younger population where the dimorphism is not very evident, there are still differences in the expected growth paths of both sexes. These have been provided by the WHO Child Growth Standards and our z-scores are calculated in accordance with them^7^. Further description of the data in this regard has been provided in the Supplementary Table S1.

After applying all of the aforementioned quality checks, we get two sets of observations—flagged and unflagged. The observations leftover after flagging can be taken to be of an acceptable quality and would thus be expected to give the true depiction of the extent of anthropometric failures. The prevalence estimates are thus calculated once before flagging and once after to ascertain the overall impact of the ‘increased quality’ of the data. The paper also presents a comparison of both groups—flagged and unflagged—in terms of their socioeconomic indicators to detect the drivers of bad quality data, if any. The variables used for the same include sex, children’s age distribution, wealth indices, and the size of the household.

It should be noted however, that deriving accurate height/length measurements for children below 24 months of age is inherently more difficult due to their method of measurement. We thus also look specifically at the age distributions pre- and post-flagging to see how the distribution changes, if at all. We revisit this issue in detail in the Discussion section. Furthermore, it is important to keep in mind that of all observations lying outside the acceptable range of our analysis, there might also be perfectly valid observations that have been ousted simply because of a lack of standardisation in the methods of measurement.

As a final part of the analysis, we seek to derive the effect of income levels on data quality. All states and union territories are classified as richer or poorer states depending on whether they are above or below the mean level of GSDP in the country. We then graph the variation in data quality over richer and poorer states and also over time. The analysis is done for both rounds of the NFHS survey. We get the data for GSDP of Indian states from the Indian Ministry of Statistics and Programme Implementation for the years 2005–2006 and 2015–2016^33^.

All analyses were performed with STATA software version 14.2.

## Conclusion

The DHS program is a global endeavor that provides crucial data on children’s health and nutrition in developing countries. Such data is used by governments and policymakers alike to track progress and plan for future policy. The estimates of stunting and underweight in particular are used to measure progress towards the Sustainable Development Goals. The quality and accuracy of such data thus become a genuine concern. The challenge lies in determining the appropriate level of data quality. This study runs a straightforward and easy-to-replicate comparative analysis of the impact of improved data quality on the prevalence estimates of stunting, underweight, and wasting in India. Our findings show that DHS data quality was found to be of an acceptable level. Further investing in improving data quality does not significantly change prevalence estimates. The age of children was also found to influence data quality, with data from younger children being less accurate than that from older ones, perhaps due to the conventional methods of measurement currently in use.

As aforementioned, DHS surveys are implemented by different nations and hence an inherent variability is bound to arise. In cases where data quality is found to be less than acceptable, improving on data quality can go a long way in making the dataset more reliable. This paper identifies data from younger children and that from poorer regions to be most prone to error. Focussing especially on these sensitive issues is one way of improving data quality. On the other hand, as long as data surveys fulfil specific quality standards, they should be considered reasonable quality and deemed reliable. Ensuring a reasonable quality of data is somewhat easier in surveys where proper technician training has been undertaken along with standardization of supervision and measurement methods as mandated by the DHS and ICF International. In such situations, data analysts can opt for more relaxed quality checks, such as choosing the more inclusive SMART SD range as opposed to the one by WHO, among others. Such practices have the effect of avoiding the loss of valuable data, while at the same time painting a more comprehensive and realistic picture of the nutritional situation in a country. In the end, allowing for reasonably relaxed quality controls will facilitate easier access to a wider array of data and freeing up resources in terms of time and money for research in novel and more pressing areas.

## Supplementary Information

Supplementary Information.

## Data Availability

The datasets analysed during the current study are available in the DHS Program repository at https://dhsprogram.com/data/dataset/India_Standard-DHS_2015.cfm?flag=0 and https://dhsprogram.com/data/dataset/India_Standard-DHS_2006.cfm?flag=1

## Abbreviations

SDG: Sustainable development goals
DHS: Demographic and Health Surveys
LMIC: Low- and middle-income countries
GSDP: Gross state domestic product
ICF: International classification of functioning, disability, and health
NFHS: National Family Health Surveys
UNICEF: United Nations International Children’s Fund
WHO: World Health Organization
SMART: Standardized monitoring and assessment of relief and transitions
SD: Standard deviations
HAZ: Height-for-age z-score
WAZ: Weight-for-age z-score
WHZ: Weight-for-height z-score

## Code availability

The STATA code used in our analysis is available upon request without restrictions.
