# Supplementary material for: The impact of improved data quality on the prevalence estimates of anthropometric measures using DHS datasets in India
Source: Sci Rep. 2021 May 21;11:10671. doi: 10.1038/s41598-021-89319-9 (PMC8140149; doi:10.1038/s41598-021-89319-9)
Supplement: Supplementary file 1 — Supplementary Information. [file 41598_2021_89319_MOESM1_ESM.docx]

Article title: The impact of improved data quality on the prevalence estimates of anthropometric measures using DHS datasets in India

Authors: HVH, DJ, RK, SV, SVS

**Supplementary**

Since the WHO Child Growth Standards are different for boys and girls, our analysis essentially controls for the age and sex of the children studied. For this purpose we present a tabulation of the age-sex specifics of our sample dataset after having dropped children under 6 months of age, missing observations, and non-singleton births. This can be found in **Supplemental Table S1**.

| Age Intervals | Male | Female |
| --- | --- | --- |
| 0-11 months | 15,072 | 13,579 |
| 12-23 months | 29,127 | 26,673 |
| 24-35 months | 28,749 | 26,562 |
| 36-47 months | 29,519 | 27,704 |
| 48-59 months | 28,711 | 26,014 |
| Total | 131,178 | 120,532 |

Table S1. Cross-tabulation of sample dataset by age interval and gender.

Supplementary Analyses:

The digit preference bias is ascertained via a visual inspection of the terminal digits of height (in cm) and that of weight (in grams). For the 10 digits from 0-9, each one is expected to have an equal distribution of 10%. Deviations from this value in excess of 2 percentage points are deemed erroneous and are thus flagged. In cases where the results of a simple visual inspection seems uncertain, we employ the Myer’s Index. The Index is based on the assumption of an equal distribution of population among different ages, and returns the deviation of the actual observations from it in percentages. Lower percentage values indicate a low digit preference bias.

**Supplemental Figure S1** plots the distribution of terminal digits for weight and height measurements in percentages. The spread of weight measurements for 2005-06 dataset is evenly distributed with values for each terminal digit close to the 10% mark. For the 2015-16 dataset however, there is a marked increase in the percentage share of the terminal digit 1 indicating a digit preference bias for rounding off weight measurements to 1 gram. Employing the Myer’s Blended Index on the same however leads to a value of 4.72 implying that 4.72% of all observations would have to be redistributed to achieve an equal distribution across all digits [17]. Since this value is still less than 5%, we have refrained from any flagging here. For the height measurements, terminal digit distributions for both datasets closely resemble a 10% share for each terminal digit. Data quality can thus be taken to be reasonably good and hence no flagging is required. As a further analysis, we still flag all observations with the terminal digit ‘1’ in weight measurements and assess its impact to ensure the robustness of our results.


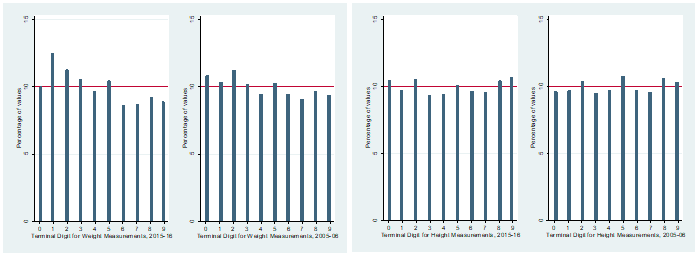
 Supplemental Figure S1. Distribution of terminal digits for weight and height measurements.

To address the effect of any potential terminal digit preference, we flag all observations with the terminal digit 1 for weight measurements in the 2015-16 dataset. The results, tabulated in **Supplemental Table S2**, show that the prevalence estimates are not significantly different from both, those prior to any flagging and those with the SMART flags for the height-for-age and weight-for-age z-score. The differences in the estimate of wasting still prevails.

|  | 2015-16 | | |
| --- | --- | --- | --- |
|  | No flags | Post flagging  (SMART) | Post flagging  (SMART and digit preference) |
| Stunting | 40.01% | 39.74% | 39.84% |
| Underweight | 36.04% | 35.72% | 35.67% |
| Wasting | 20.59% | 18.03% | 17.76% |

Supplemental Table S2. Prevalence estimates of anthropometric failures before and after flagging.

Incidentally, generating another flag specifically for the WHZ of the 2015-16 dataset to arrive at a lower SD, we get an even lower estimate of wasting. Restricting the range of acceptable observations to those having a WHZ of more than -4.5 and less than 0.9 (-4.5<WHZ<0.9), the estimate of wasting falls only slightly from 20.59% to 19.65%. The SD of the WHZ is now reported at 1.10, which fits the acceptable SD range for both the WHO and SMART. Note that this range is only slightly more inclusive on the lower end of the distribution than the one used earlier (-4.50 as opposed to the former -4.09), but more restrictive on the upper end (0.9 as opposed to the former 2.10). This goes to show the sensitivity of the SMART flags to the means of a distribution and their significance in the eventual calculation of prevalence estimates.
